# Changes in the proportion and severity of patients with fever or common cold symptoms utilizing an after-hours house call medical service during the COVID-19 pandemic in Tokyo, Japan: a retrospective cohort study

**DOI:** 10.1186/s12873-021-00458-8

**Published:** 2021-05-29

**Authors:** Ryota Inokuchi, Kojiro Morita, Masao Iwagami, Taeko Watanabe, Masatoshi Ishikawa, Nanako Tamiya

**Affiliations:** Department of Health Services Research, Faculty of Medicine, University of Tsukuba, 1-1-1 Tenno-dai, Tsukuba, Ibaraki, 305-8575 Japan

**Keywords:** Out-of-hours primary care, Out-of-hours service, Quality, Severe acute respiratory syndrome, Severity, Triage

## Abstract

**Background:**

Trends in the characteristics and disease severity of patients using an after-hours house call (AHHC) medical service changed during the coronavirus disease (COVID-19) pandemic. However, there have been no reports on this issue since the start of the COVID-19 pandemic. This study aimed to investigate patients’ tendencies to utilize an AHHC medical service for fever or common cold symptoms during the COVID-19 pandemic.

**Methods:**

This retrospective cohort study compared the characteristics and disease severity of patients with fever or common cold symptoms utilizing an AHHC medical service offered by a single large company between the control period (December 1, 2018 to April 30, 2019) and the COVID-19 pandemic exposure period (December 1, 2019 to April 30, 2020). We also assessed the proportion of these patients in relation to all patients calling the service for any reason.

**Results:**

During the control and COVID-19 pandemic exposure periods, a total of 6462 and 10,003 patients consulted the AHHC medical service, respectively. Of these, 5335 (82.6%) and 7423 (74.2%) patients had fever and common cold symptoms, respectively, during the control and COVID-19 pandemic exposure periods (*P* < 0.001). The corresponding median (interquartile range) ages were 8 (3–11) and 10 (4–33) years, respectively. The distribution of disease severity differed between the groups. The proportions of patients with mild, moderate, and severe illness were 71.1, 28.7, and 0.2% in the control period and 42.3, 56.7, and 0.9% in the COVID-19 pandemic exposure period, respectively (*P* < 0.001).

**Conclusions:**

During the COVID-19 pandemic, the proportion of patients with fever or common cold symptoms was lower than that in the control period, but disease severity was significantly higher.

## Background

Since the identification of severe acute respiratory syndrome coronavirus 2 (SARS-CoV-2) at the end of 2019, the coronavirus disease (COVID-19) pandemic has resulted in a global health crisis [1]. Since January 2020, the number of COVID-19 cases has markedly increased in Japan, particularly in Tokyo [2, 3]. However, at the beginning of the COVID-19 pandemic, many health care providers in clinics and general hospitals were reluctant to examine patients with fever or common cold symptoms, mainly due to difficulties in ensuring proper infection control measures, such as distancing of outpatients or the lack of personal protective equipment (PPE) for health care providers. This placed an additional burden on emergency departments (EDs) [4].

A decade before the COVID-19 pandemic, ED overcrowding had become a significant public health problem worldwide [5]. Previous studies have reported that not all patients necessarily require an ambulance to reach the ED [6], and some reports have shown that 30–80% of patients can be appropriately treated in primary care settings [7, 8]. To reduce unnecessary ambulance transport, and thereby reduce ED overcrowding, many countries have recently launched after-hours house call (AHHC) medical services or out-of-hours (OOH) services [9].

In Tokyo, a private, non-governmental emergency medical service, which sends doctors directly to patients’ residences who need medical attention via telephone triage, has been operational since 2016. In Japan, generally, when a complaint arises OOH and patients are unable to arrange for a house doctor, they usually call an ambulance directly, call an emergency telephone consultation service, call a home clinic or hospital, directly make a walk-in visit to an ED, or wait until a hospital opens. Emergency hospitals in Japan are divided into primary, secondary, and tertiary hospitals. When an ambulance is called, patients are transferred to a secondary or tertiary hospital, depending on the degree of severity. In contrast, ambulatory patients can freely consult a primary hospital, a secondary hospital, or in some cases, a tertiary hospital directly [10].

We hypothesized that the proportion and severity of patients with fever or common cold symptoms using an AHHC medical service changed during the COVID-19 pandemic. To our knowledge, there have been no reports on whether AHHC medical services have reduced the burden on EDs since the start of the COVID-19 pandemic. Therefore, this study aimed to investigate patients’ tendencies to utilize an AHHC medical service for fever or common cold symptoms between the control period and COVID-19 pandemic exposure period.

## Methods

We compared the characteristics of patients using the AHHC medical service in Japan for complaints of fever or common cold symptoms between the COVID-19 pandemic exposure period and the same period in the previous year (control period).

### Study design

This study was a retrospective study using anonymized data from medical records of patients using the AHHC medical service from December 1, 2018 to April 30, 2020. The study design was reviewed and approved by the Research Ethics Committee of the University of Tsukuba (approval number: 1527).

### After-hours house call (AHHC) medical service in Japan

Since 2016 in Tokyo, Fast Doctor Ltd. (Shinjuku, Tokyo, Japan) has been running a private AHHC medical service, which sends doctors directly to a patient’s residence instead of sending an ambulance. The company operates 7 days a week, outside of regular hospital visiting hours (19:00–06:00 on weekdays, 18:00–06:00 on Saturdays, and 24 h a day on Sundays and holidays). The AHHC medical service has adopted a telephone triage approach. A well-used telephone triage in Japan is as follows: when a patient calls an emergency telephone consultation service, an operator classifies the patient into one of five categories (red, orange, yellow, green, or white) based on acuity. The action for consultation is as follows: red, call an ambulance and transport the patient to a secondary or tertiary emergency hospital; orange, provide the patient with information about a nearby secondary emergency hospital; yellow and white, provide the patient with information about nearby clinics or a primary hospital; and white, provide the patient with advice for home observation [11].

AHHC doctors also conduct home visits for patients classified as orange and yellow, and following consultation, the doctor assesses the severity (mild, moderate, or severe) of the illness. Disease severity is classified as follows: mild, the patient can use over-the-counter drugs; moderate, the patient needs to visit a hospital or clinic; and severe, an ambulance is needed. The number of visiting doctors per shift is four to twelve.

Before the COVID-19 pandemic, AHHC medical service operators triaged patients with fever or common cold symptoms via telephone. After the start of the COVID-19 pandemic, operators assessed risk factors for COVID-19, in addition to performing the telephone triage. They also advised patients to call the consultation service in public health centers if COVID-19 was suspected, according to the Japanese government guidelines [12].

**Fig. 1:**
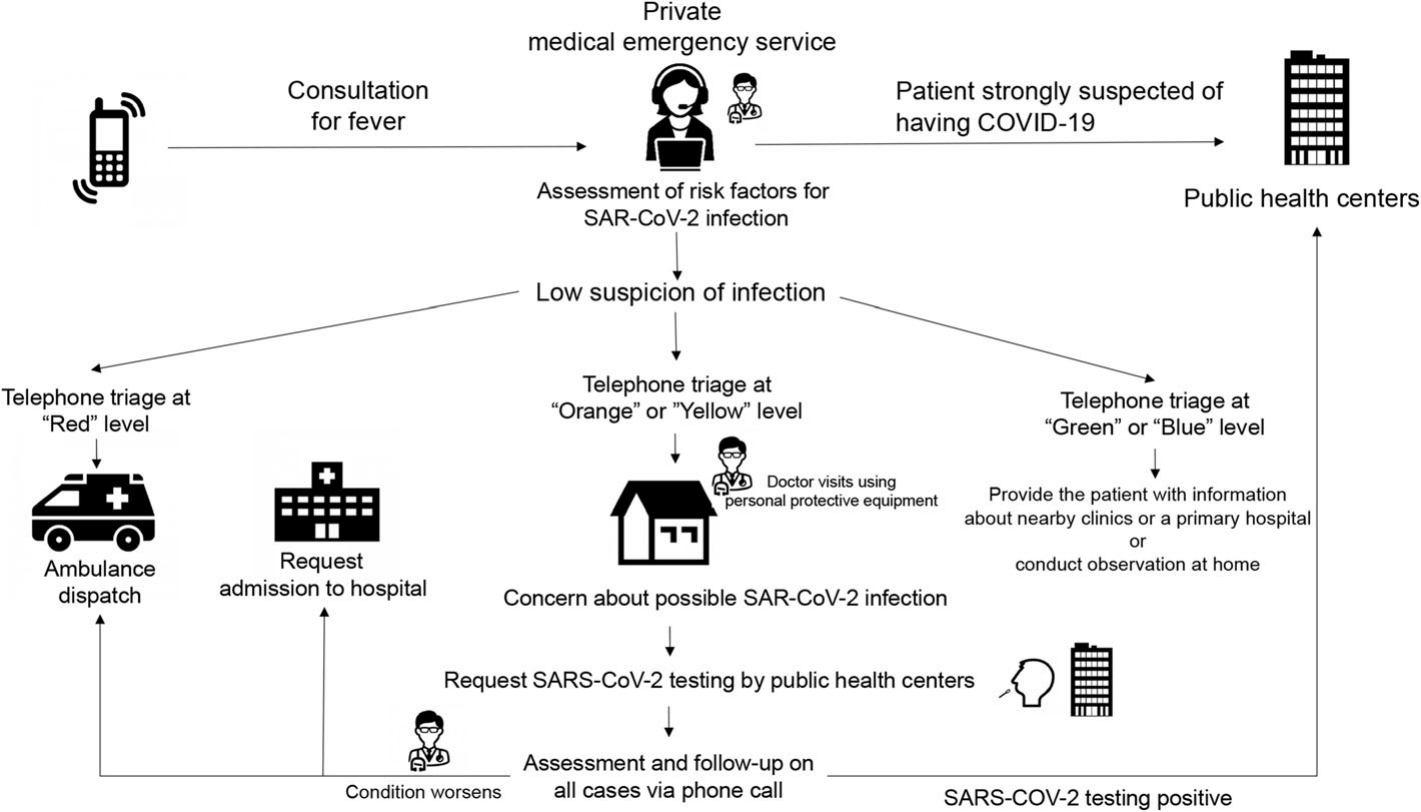
Flow chart of patients who consulted the after-hours house call medical service The operators of the private medical emergency service assess risk factors for severe acute respiratory syndrome coronavirus 2 (SARS-CoV-2) infection. If infection is suspected, the operators consult a doctor and advise the patients to call a public health center. Then, based on the telephone triage (red, orange, yellow, green, and white), the operators determine whether the patient is to remain at home (white), or they opt to provide the patient with information about nearby clinics or a primary hospital (green), a doctor’s visit to the patient’s residence (orange and yellow), or an ambulance (red)

When there was a low suspicion of COVID-19, doctors visited patients with fever at home, if their triage level was orange or yellow. The visiting doctor used PPE while examining the patient and requested the SARS-CoV-2 polymerase chain reaction test from a public health center, if COVID-19 was suspected. All patients with fever were followed-up with a daily phone call until their symptoms improved. When patients tested positive for COVID-19, the AHHC medical service informed the public health centers and established contact with the patients. If the patient’s condition deteriorated during the follow-up period, the visiting doctor assessed the severity and determined whether the patient should continue to be isolated at home, request admission to a hospital, or call an ambulance, regardless of their SARS-CoV-2 test results (Fig. 1).

### Setting and participants

All patients using the AHHC medical service during the control period (December 1, 2018 to April 30, 2019) and COVID-19 pandemic exposure period (December 1, 2019 to April 30, 2020) were included in this study. During each study period, we first identified patients calling the AHHC medical service for any reason; this number was used as the denominator. Among them, we identified patients with a fever, defined as a body temperature ≥ 37.5 °C according to the infectious disease control law in Japan, as well as those with common cold symptoms as the main population of interest in this study.

### Variables

The following data of patients with fever or common cold symptoms were extracted: sex, age, comorbidities (hypertension, diabetes mellitus, hyperlipidemia, gout, chronic lung disease, heart failure, liver disease, cerebral infarction, cancer, collagen disease, and dementia), diagnosis, and disease severity.

### Data source

The study used anonymized data from the medical records of patients using the AHHC medical service. These data were provided to the study group by Fast Doctor Ltd. To compare the characteristics of patients with a fever or common cold symptoms between the control period (December 1, 2018 to April 30, 2019) and COVID-19 pandemic exposure period (December 1, 2019 to April 30, 2020), the records of all patients who consulted Fast Doctor Ltd. during these periods were reviewed.

### Statistical analyses

First, we examined the number of patients with fever or symptoms of the common cold, and its proportion among all patients calling the AHHC medical service (for any reason) between the COVID-19 pandemic exposure period and control period. Second, we compared the characteristics (age, sex, comorbidities, number of patients per month, diagnosis, and disease severity) of patients with fever or common cold symptoms using the chi-square test. Third, we stratified age (< 15, 15–64, and ≥ 65 years) and compared the severity level of each period using the chi-square test. Analyses were performed using JMP 14.3 statistical software (SAS Institute Inc., Cary, NC, USA). The significance threshold was set at *P* < 0.05.

## Results

### Proportion and basic characteristics of patients with fever or common cold symptoms

A total of 6739 patients in the control period and 10,008 patients in the COVID-19 pandemic exposure period consulted the AHHC medical service, respectively. We excluded 277 and five patients from the control and COVID-19 pandemic exposure periods, respectively, due to a lack of data on age, sex, and telephone triage, leaving a total of 6462 and 10,003 patients utilizing the AHHC medical service. Of these, 5335 (82.6%) and 7423 (74.2%) patients had fever or common cold symptoms in the control period and COVID-19 pandemic exposure period, respectively (*P* < 0.001); their details are shown in Table 1.

**Table 1:** Characteristics of patients with fever for each period

Characteristic	Control period (*n* = 5335)	Exposure period (*n* = 7423)	*P*-value
Age, years			
Median (IQR)	8 (3–11)	10 (4–33)	< 0.001
Category, n (%)			< 0.001
0–14	3147 (59.0)	4152 (55.9)	
15–64	2112 (39.6)	3089 (41.6)	
≥ 65	76 (1.4)	182 (2.5)	
Male sex, n (%)	2765 (48.2)	3839 (48.3)	0.90
Month, n (%)			< 0.001
December	523 (9.8)	1683 (22.7)	
January	1704 (31.9)	2052 (27.6)	
February	1243 (23.3)	2062 (27.8)	
March	900 (16.9)	1057 (14.2)	
April	968 (18.1)	570 (7.7)	
Diagnosis, n (%)			
Upper respiratory tract infection	4555 (85.4)	5875 (79.1)	< 0.001
Influenza	1916 (35.9)	2119 (28.5)	< 0.001
Streptococcal infection	78 (1.5)	161 (2.2)	< 0.001
Tonsillitis	191 (3.6)	254 (3.4)	0.63
Sinusitis	15 (0.3)	34 (0.5)	0.11
COVID-19	0 (0.0)	6 (0.1)	0.038
Pneumonia	38 (0.7)	61 (0.8)	0.49
Asthma	48 (0.9)	40 (0.5)	0.021
Otitis media	72 (1.4)	106 (1.4)	0.71
Urinary tract infection	24 (0.4)	67 (0.9)	< 0.001
Gastroenteritis	773 (14.5)	1064 (14.3)	0.22
Other abdominal disease	35 (0.7)	82 (1.1)	< 0.001
Other	138 (2.6)	427 (5.7)	< 0.001
Comorbidities, n (%)			
Hypertension	28 (0.5)	65 (0.9)	0.019
Diabetes mellitus	21 (0.4)	40 (0.5)	0.24
Hyperlipidemia	10 (0.2)	17 (0.2)	0.61
Gout	3 (0.1)	5 (0.1)	0.80
Chronic lung disease	178 (3.3)	230 (3.1)	0.45
Heart failure	0 (0)	5 (0.1)	0.06
Liver disease	6 (0.1)	14 (0.2)	0.27
Cerebral infarction	8 (0.2)	14 (0.2)	0.60
Cancer	30 (0.6)	59 (0.8)	0.12
Collagen disease	5 (0.1)	6 (0.1)	0.27
Dementia	3 (0.1)	7 (0.1)	0.45

*COVID-19* coronavirus disease; *IQR* interquartile range

The median (interquartile range) ages of patients in the control and COVID-19 pandemic exposure periods were 8 (3–11) and 10 (4–33) years, respectively. Patients aged < 15 years (59.0 and 55.9%, respectively) and those aged 15–64 years (39.6 and 41.6%, respectively) comprised the majority of users of the AHHC medical service both in the control and COVID-19 pandemic exposure periods. After stratifying the patients into three age groups (in both periods), the proportion of patients aged ≥65 years with fever or common cold symptoms was higher during the COVID-19 pandemic exposure period than during the control period (2.5% vs. 1.4%). There were no differences in sex ratio or comorbidities (except hypertension) between the two groups. The number and proportion of patients with fever or common cold symptoms declined sharply in April 2020 (Table 1).

### Causes of fever

Most diagnoses were upper respiratory tract infections, including influenza, streptococcal infections, tonsillitis, and sinusitis (Table 1). Among upper respiratory tract infections, the proportions of influenza infections and asthma were significantly lower, while those of streptococcal and urinary tract infections were significantly higher during the COVID-19 pandemic exposure period than during the control period. During the COVID-19 pandemic exposure period, the AHHC medical service performed SARS-CoV-2 tests on 12 patients, six of whom had positive test results (Table 1); an ambulance was dispatched for one patient, while the other five requested hospital admission.

### Comparison of disease severity in each period

The proportion of severe patients with fever or common cold symptoms increased (0.2 and 0.9%), and that of patients assessed to have moderate severity nearly doubled during the pandemic exposure period compared with that in the control period (56.7% vs. 28.7%; Table 2). After stratifying the patients into three age groups, all age groups also showed severity that significantly increased in the exposure period compared to that in the control period (*p* < 0.001).

**Table 2:** Comparison of the severity level in each period according to age

Severity	Control period (*n* = 5335)	Exposure period (*n* = 7423)	*P-*value
All			
Severity level			< 0.001
1 (severe)	12 (0.2)	69 (0.9)	
2 (moderate)	1531 (28.7)	4212 (56.7)	
3 (mild)	3792 (71.1)	3142 (42.3)	
Age < 15 years			
Severity level			< 0.001
1 (severe)	5 (0.2)	13 (0.3)	
2 (moderate)	855 (27.1)	2297 (55.3)	
3 (mild)	2287 (72.7)	1842 (44.4)	
Age 15–64 years			
Severity level			< 0.001
1 (severe)	5 (0.2)	30 (1.0)	
2 (moderate)	652 (30.9)	1821 (59.0)	
3 (mild)	1495 (68.9)	1238 (40.1)	
Age ≥ 65 years			
Severity level			< 0.001
1 (severe)	2 (2.7)	26 (14.3)	
2 (moderate)	24 (31.6)	94 (51.7)	
3 (mild)	50 (65.8)	62 (34.1)	

## Discussion

### General findings

In this study, we found that the proportion of patients with fever or common cold symptoms using the AHHC medical service in Japan was slightly lower, but the severity of patients was substantially higher, in the COVID-19 pandemic exposure period than in the control period.

### Reduction in the proportion of patients with fever or common cold symptoms

The proportion of patients with fever or common cold symptoms was lower during the COVID-19 pandemic exposure period than during the control period. We had initially hypothesized that patients with fever or common cold symptoms may be reluctant to visit hospitals due to the risk of cross-infection and may have been concerned about which hospitals to approach; this would have increased the demand for the AHHC medical service after the start of the pandemic. However, the proportion of patients with fever or common cold symptoms using the AHHC medical service clearly decreased.

The reason for this result is unclear. At the beginning of the COVID-19 pandemic in Japan, patients with (1) a cold or fever of ≥37.5 °C lasting for ≥4 days and (2) intense malaise or dyspnea were advised to consult a public health center, as per the Japanese government guidelines. Thus, patients with fever or common cold symptoms may have refrained from visiting a hospital or clinic, leading to a decrease in the number of patients in these medical settings. These patients may also have refrained from using an AHHC medical service.

The proportion of patients with fever or common cold symptoms decreased sharply in April 2020. The reasons for this finding may be as follows: (1) the Japanese government declared the temporary closure of all Japanese elementary, junior high, and high schools at the end of February [13]; and (2) on April 7, 2020, a state of emergency was initially declared in seven prefectures, including Tokyo. Thereafter, many companies requested their employees to work from home, conducted meetings remotely, and restricted entry to the office [14]. This resulted in lower seasonal influenza activity in 2020 than in previous years [15].

### Increase in the proportion of patients with severe illness

In contrast, the proportion of patients with more severe conditions was higher in the COVID-19 pandemic exposure period than in the control period. Recently, several studies have raised concerns about the increased risk of delays in receiving medical care due to restraint from going to the hospital during the COVID-19 pandemic exposure period [16–19]. One reason was the fear of cross-infection in the community and hospital. However, patients could avoid cross-infection by calling the AHHC medical service. Further research is needed to determine whether government policies increase the severity of patients due to restraint from hospital and AHHC medical service consultations.

Patients with COVID-19 can show progression to respiratory failure within hours [20–22]; in particular, older age and chronic medical conditions have been associated with higher mortality [23–27]. Since early detection is crucial for older patients with fever or common cold symptoms, use of the AHHC medical service may contribute to early detection and reduce the patient’s hesitancy to call an ambulance.

### Reduction of the burden on EDs, public health centers, and clinics

The AHHC medical service has provided > 7000 medical consultations since the start of the pandemic. On April 20, 2020, nine prefectures, including Tokyo, reported that their hospitals were already at 80% capacity, and a public broadcaster in Japan reported that a patient with COVID-19 symptoms had been turned away by 80 hospitals in Tokyo [3]. Outpatient management is appropriate for most patients with COVID-19. In approximately 80% of patients, the illness is mild and does not warrant medical intervention or hospitalization [28]. Thus, the AHHC medical service may have acted as a buffer for consultations for patients with suspected COVID-19.

In addition, the AHHC medical service may reduce the burden on public health centers, clinics, and patients due to the AHHC process, home visits, and direct consultations with patients at home, thereby contributing to a reduction in the risk of cross-infection in the community and hospital. This information may help drive health care policy development and social behaviors when a new pandemic occurs in the future, to reduce the burden on EDs and public health centers by using the AHHC medical service.

### Limitations

There were some limitations to this study. First, we reported only a single AHHC medical service in Japan, resulting in potential selection bias. However, this AHHC medical service provides > 18,000 night visits annually and is the largest OOH emergency service in Japan. Second, we only compared two periods. After April 10, 2020, the Japanese government lifted restrictions on first-time patients online or via telephone; thus, patients no longer needed to visit a hospital or clinic. In addition, telemedicine has been covered by Japan’s National Health Insurance program. The medical situation has changed markedly; thus, we only compared patients from December to April for each period. Finally, the AHHC medical service has a low usage rate among elderly people who may not be familiar with its use as the AHHC medical service was introduced in 2016.

## Conclusion

At the beginning of the COVID-19 pandemic, the severity of patients with fever or common cold symptoms using the AHHC medical service was higher than that in the control period. Further research is needed to determine whether the increase in the severity of patients was a result of restraint from consultations.

## Data Availability

The datasets used and/or analyzed during the current study are available from the corresponding author on reasonable request.

## Abbreviations

AHHC: After-hours house call
COVID-19: Coronavirus disease 2019
ED: Emergency department
OOH: Out-of-hours
PPE: Personal protective equipment; severe acute respiratory syndrome coronavirus 2

## References

[1] Zhu N, Zhang D, Wang W, Li X, Yang B, Song J, Zhao X, Huang B, Shi W, Lu R, Niu P, Zhan F, Ma X, Wang D, Xu W, Wu G, Gao GF, Tan W, China Novel Coronavirus Investigating and Research Team (2020). A novel coronavirus from patients with pneumonia in China, 2019. N Engl J Med.

[2] Looi MK (2020). Covid-19: Japan declares state of emergency as Tokyo cases soar. BMJ..

[3] Hayasaki E (2020). Covid-19: how Japan squandered its early jump on the pandemic. BMJ..

[4] Nakahara S, Kanda J, Miyake Y, Sakamoto T (2021). High incidence of heat illness and the potential burden on the health care system during the COVID-19 pandemic. Lancet Reg Health West Pac.

[5] Hoot NR, Aronsky D (2008). Systematic review of emergency department crowding: causes, effects, and solutions. Ann Emerg Med.

[6] Booker MJ, Shaw AR, Purdy S (2015). Why do patients with ‘primary care sensitive’ problems access ambulance services? A systematic mapping review of the literature. BMJ Open.

[7] Gratton MC, Ellison SR, Hunt J, Ma OJ (2003). Prospective determination of medical necessity for ambulance transport by paramedics. Prehosp Emerg Care.

[8] Patton GG, Thakore S (2013). Reducing inappropriate emergency department attendances--a review of ambulance service attendances at a regional teaching hospital in Scotland. Emerg Med J.

[9] Australian Government Department of Health. Review of after hours primary health care. 2015. http://www.health.gov.au/internet/main/publishing.nsf/Content/primary-ahphc-review. Accessed 1 November 2020.

[10] Inokuchi R, Sato H, Nakajima S, Shinohara K, Nakamura K, Gunshin M, Hiruma T, Ishii T, Matsubara T, Kitsuta Y, Yahagi N (2013). Development of information systems and clinical decision support systems for emergency departments: a long road ahead for Japan. Emerg Med J.

[11] Sakurai A, Morimura N, Takeda M, Miura K, Kiyotake N, Ishihara T, Aruga T (2014). A retrospective quality assessment of the 7119 call triage system in Tokyo - telephone triage for non-ambulance cases. J Telemed Telecare.

[12] Ministry of Health, Labour and Welfare. Basic policies for novel coronavirus disease control. 2020. https://www.mhlw.go.jp/content/10900000/000620733.pdf. Accessed 1 November 2020.

[13] Amengual O, Atsumi T (2021). COVID-19 pandemic in Japan. Rheumatol Int.

[14] U.S. Embassy & Consulates in Japan. Health alert – U.S. Embassy Tokyo. 2020. https://jp.usembassy.gov/health-alert-us-embassy-tokyo-april29-2020/. Accessed 1 November 2020.

[15] Sakamoto H, Ishikane M, Ueda P (2020). Seasonal influenza activity during the SARS-CoV-2 outbreak in Japan. JAMA..

[16] Lazzerini M, Barbi E, Apicella A, Marchetti F, Cardinale F, Trobia G (2020). Delayed access or provision of care in Italy resulting from fear of COVID-19. Lancet Child Adolesc Health.

[17] Jeffery MM, D’Onofrio G, Paek H, Platts-Mills TF, Soares WE, Hoppe JA (2020). Trends in emergency department visits and hospital admissions in health care systems in 5 states in the first months of the COVID-19 pandemic in the US. JAMA Intern Med.

[18] Kastritis E, Tsitsimpis K, Anninos E, Stamatelopoulos K, Kanakakis I, Lampropoulos C (2020). Significant reduction in the visits to the emergency room department during the COVID-19 pandemic in a tertiary hospital in Greece: indirect victims of the pandemic?. Med (Baltim).

[19] Lynn RM, Avis JL, Lenton S, Amin-Chowdhury Z, Ladhani SN (2021). Delayed access to care and late presentations in children during the COVID-19 pandemic: a snapshot survey of 4075 paediatricians in the UK and Ireland. Arch Dis Child.

[20] Chan JF, Yuan S, Kok KH, To KK, Chu H, Yang J (2020). A familial cluster of pneumonia associated with the 2019 novel coronavirus indicating person-to-person transmission: a study of a family cluster. Lancet..

[21] Phan LT, Nguyen TV, Luong QC, Nguyen TV, Nguyen HT, Le HQ (2020). Importation and human-to-human transmission of a novel coronavirus in Vietnam. N Engl J Med.

[22] Burki TK (2020). Coronavirus in China. Lancet Respir Med.

[23] Lighter J, Phillips M, Hochman S, Sterling S, Johnson D, Francois F, Stachel A (2020). Obesity in patients younger than 60 years is a risk factor for COVID-19 hospital admission. Clin Infect Dis.

[24] Richardson S, Hirsch JS, Narasimhan M, Crawford JM, McGinn T, Davidson KW, Barnaby DP, Becker LB, Chelico JD, Cohen SL, Cookingham J, Coppa K, Diefenbach MA, Dominello AJ, Duer-Hefele J, Falzon L, Gitlin J, Hajizadeh N, Harvin TG, Hirschwerk DA, Kim EJ, Kozel ZM, Marrast LM, Mogavero JN, Osorio GA, Qiu M, Zanos TP, and the Northwell COVID-19 Research Consortium (2020). Presenting characteristics, comorbidities, and outcomes among 5700 patients hospitalized with COVID-19 in the new York City area. JAMA..

[25] Docherty AB, Harrison EM, Green CA, Hardwick HE, Pius R, Norman L (2020). Features of 20 133 UK patients in hospital with covid-19 using the ISARIC WHO clinical characterisation protocol: prospective observational cohort study. BMJ..

[26] Zhou F, Yu T, Du R, Fan G, Liu Y, Liu Z (2020). Clinical course and risk factors for mortality of adult inpatients with COVID-19 in Wuhan, China: a retrospective cohort study. Lancet..

[27] Onder G, Palmieri L, Vanacore N, Giuliano M, Brusaferro S, Italian National Institute of Health COVID-19 Mortality Group (2021). Nonrespiratory complications and obesity in patients dying with COVID-19 in Italy. Obesity (Silver Spring).

[28] World Health Organization. Report of the WHO-China joint mission on coronavirus disease 2019 (COVID-19). 2020. https://www.who.int/publications-detail/report-of-the-who-china-joint-mission-on-coronavirus-disease-2019-(covid-19). Accessed 1 November 2020.

